# Finite element stress analysis to evaluate and compare the effect of splinting in periodontally compromised teeth having 55 % bone loss with different materials

**DOI:** 10.1016/j.jobcr.2025.01.011

**Published:** 2025-02-10

**Authors:** Shivangi Joshi, Prakash Talreja, Prajakta Rao, Nilesh Joshi, Vinayak Thorat, Ruhi Mohta

**Affiliations:** Department of Periodontology, Bharati Vidyapeeth Dental College and Hospital, Navi Mumbai, India

**Keywords:** Finite element analysis, Periodontally compromised teeth, Splinting materials, Polyetheretherketone (PEEK), Fiber-reinforced composite (FRC), Stress distribution, Von mises stress

## Abstract

**Background:**

Splinting is commonly employed to stabilize periodontally compromised teeth, but the choice of splint material significantly influences stress distribution within the periodontal ligament (PDL) and surrounding bone. This study aimed to evaluate and compare the stress distribution of four different splint materials—composite, fiber-reinforced composite (FRC), polyetheretherketone (PEEK), and metal—on mandibular anterior teeth with 55 % bone loss using finite element analysis (FEA).

**Methodology:**

Finite element models of mandibular anterior teeth with 55 % bone loss were developed using SOLIDWORKS 2020. Simulations were conducted for non-splinted teeth and for teeth splinted with composite, FRC, PEEK, and metal. Stress analysis was performed in ANSYS software under vertical (100N at 0°) and oblique (100N at 45°) loading conditions. Von Mises stress values in the PDL and cortical bone were recorded and statistically analyzed using MedCalc software to compare the performance of different splint materials.

**Results:**

Non-splinted teeth exhibited the highest stress levels, particularly under oblique loading conditions. Among the splinting materials, FRC showed the most effective reduction in stress across all teeth, especially under vertical loads. Composite and metal wire splints provided moderate stress reduction, with performance varying depending on the load angle. PEEK splints demonstrated good stress reduction under vertical loads but showed increased stress levels under oblique forces. These results underscore the influence of splint material and load direction on stress distribution in periodontally compromised teeth.

**Conclusion:**

The study highlights the critical role of splint material in reducing stress on the PDL of periodontally compromised teeth. FRC splints emerged as the most effective material for minimizing stress under both vertical and oblique loading conditions. Composite and metal wire splints offered moderate efficacy, while PEEK splints were less effective under oblique loads. These findings provide valuable insights for clinicians in selecting optimal splint materials for periodontal stabilization.

## 1 Introduction

Management of periodontally compromised teeth is a significant challenge in modern dentistry, particularly when extensive bone loss is involved.^1^ Periodontal diseases, characterized by the progressive loss of alveolar bone and periodontal attachment, lead to weakening of tooth support structures, necessitating various restorative interventions.^2^

Splinting is a well-established technique used to stabilize periodontally compromised teeth, aiming to distribute occlusal forces more evenly and reduce stress on individual teeth.^3^ The choice of splint material is crucial because different materials exhibit varying mechanical properties that influence their effectiveness in stress distribution.^4^ Composite materials, fiber-reinforced composites, polyetheretherketone (PEEK), and metals are commonly used in splint fabrication, each offering distinct advantages and limitations.^5^

Composite materials are popular owing to their ease of use and adaptability to tooth contours. However, their long-term effectiveness in severe periodontal cases has been debated, and fiber-reinforced composites provide enhanced strength and durability, making them suitable for more demanding applications.^5^ PEEK, known for its biocompatibility and high mechanical strength, is a promising alternative to traditional materials.^6^ Metal splints, although less flexible, offer superior mechanical properties and have been the gold standard in many cases.^7^

Finite Element Analysis (FEA) has emerged as a powerful tool for evaluating the biomechanical behavior of dental restorations and splints.^8^ By simulating real-world conditions and analyzing the stress distribution, FEA provides valuable insights into the performance of different splint materials under simulated clinical conditions.^9^ Previous studies have demonstrated the efficacy of FEA in assessing stress distribution in various dental applications, including implant-supported restorations and endodontic treatments.^10^

This study aimed to evaluate and compare the stress distributions of four different splint materials—composite, fiber-reinforced composite, polyetheretherketone (PEEK), and metal—applied to periodontally compromised teeth with 55 % bone loss in the anterior region. The primary objective was to assess how each splint material influenced the stress distribution in these severely affected teeth. Additionally, this study sought to compare the effectiveness of these splint materials to determine which provided the best stability and stress management. To achieve this, the study tested two hypotheses: the null hypothesis (H₀) posited that there was no significant difference in stress distribution among the four splint types under two different loading angles, while the alternate hypothesis (H₁) suggested that there was a significant difference in stress distribution among at least two of the four splint types when subjected to varying loading angles.

## 2 Materials and methods

### 2.1 Source of data

The finite element models utilized in this study were meticulously prepared by Engineering DNA, a specialized center with expertise in creating advanced simulation models. The center designed and provided models that were subjected to simulated force variations to evaluate and compare the stress distribution around the periodontally compromised teeth using different splint materials.

### 2.2 Method of data collection

Inclusion Criteria: The study focused on the mandibular anterior region, which is characterized by type II bone density. This region was selected to ensure consistency in bone density and periodontal compromise across the study.

### 2.3 Study design and methodology

This study employed a detailed finite element analysis (FEA) approach to assess and compare the stress distributions of four different splint materials. The construction of the finite element model involved several key steps, each designed to ensure accurate representation and analysis of splint material performance. The methodology is outlined below in detail.

#### 2.3.1 Construction of 3D models

The three-dimensional (3D) models of mandibular anterior teeth were constructed using SOLIDWORKS 2020, a sophisticated CAD software known for its precision and intricate modeling capabilities. The study evaluated four splint types categorized into the following groups: composite (Group A), fiber-reinforced composite (FRC; Group B), polyetheretherketone (PEEK; Group C), and metal (Group D). Composite splints were chosen for their ease of use and adaptability, while FRC splints were selected for their enhanced strength and durability. PEEK splints, recognized for their mechanical strength and biocompatibility, represented a high-performance polymer option. Metal splints, made from various alloys, were included for their superior mechanical properties. All splint models adhered to standardized design specifications, including uniform dimensions, geometric features, and material thicknesses, as outlined by the primary investigator to ensure consistency across all groups.

#### 2.3.2 Finite element model preparation

The finite element models underwent meshing, a process that discretized the 3D structures into smaller elements for stress analysis. ANSYS software was used to create a highly refined mesh, enabling accurate capture of stress variations across the models. Each splint material was assigned specific mechanical properties, such as Young's modulus, density, and Poisson's ratio, derived from standard data sources to simulate realistic conditions. Boundary conditions were then applied to define the interaction of the splints with their environment. Simulated forces, including vertical (100N at 0°) and oblique (100N at 45°) loads, were applied to replicate clinical scenarios.

#### 2.3.3 Simulation and stress analysis

Finite element analysis simulations were performed using ANSYS software to calculate the stress distribution across the splint models. The Von Mises stress criterion, commonly used to predict material failure under complex loading conditions, was employed to evaluate the stress distribution in the periodontal ligament (PDL) and cortical bone.

#### 2.3.4 Post-processing and results evaluation

Post-simulation, the stress distribution results were analyzed to compare the performance of the four splint materials under vertical and oblique loading conditions. These findings provided insights into the effectiveness of each splint type in managing stress, facilitating an evidence-based assessment of their suitability for stabilizing periodontally compromised teeth.

### 2.4 Statistical analysis

The statistical analysis of the study involved several critical steps to ensure the accurate processing and interpretation of data from Finite Element Analysis (FEA) simulations. Initially, the data were entered into Microsoft Excel for organization and validation, with rigorous checks for errors and consistency. The validated data were then analyzed using MedCalc Statistical Software, where descriptive statistics, comparative analyses (such as ANOVA or Kruskal-Wallis tests), and post-hoc testing were performed to assess differences in stress distribution among splint materials. Statistical significance was determined using p-values, typically set at 0.05, and the results were visualized using graphs and charts.

## 3 Results

**Fig. 1:**
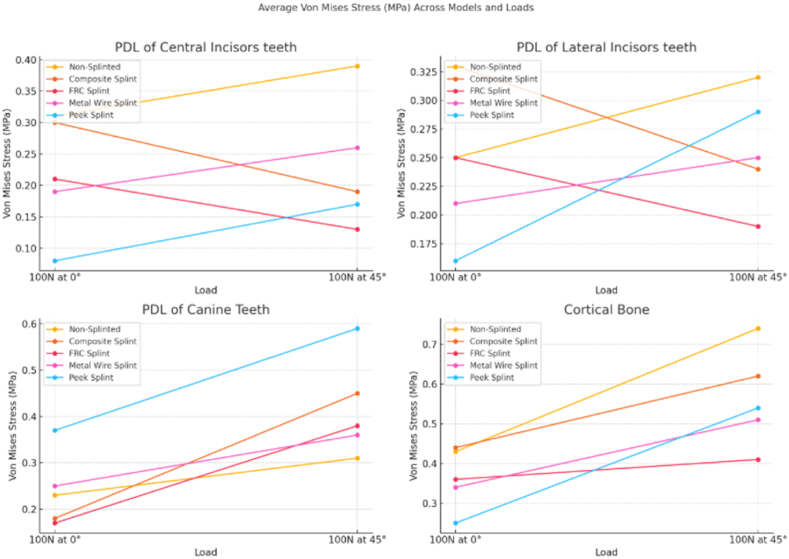
Average Von Mises stress (MPa) for each dental part (PDL of Central Incisors, Lateral Incisors, Canine Teeth, and Cortical Bone) across different models and loads.

The analysis of average Von Mises stress, measured in MPa, highlights the impact of splinting on the periodontal ligament (PDL) and cortical bone under varying loading conditions. For the non-splinted model under a 100N vertical load at 0°, the stress values observed in the PDL of the central incisors, lateral incisors, and canine teeth were 0.31, 0.25, and 0.23 MPa, respectively, while the cortical bone exhibited a stress value of 0.43 MPa. However, when the load was applied at an oblique angle of 45°, there was a significant increase in stress levels, particularly in the cortical bone, which rose from 0.43 to 0.74 MPa. These findings emphasize the role of loading direction in influencing stress distribution and underscore the necessity of splinting to mitigate stress in periodontally compromised teeth (Table 1, Fig. 1).

**Table 1:** Average von mises stress (MPa).

AVERAGE VON MISES STRESS (Mpa)					
Model	LOAD (N)	PDL of Central Incisors teeth	PDL of Lateral Incisors teeth	PDL of Canine Teeth	Cortical Bone
**Non-Splinted**	100N at 0°	0.31	0.25	0.23	0.43
	100N at 45°	0.39	0.32	0.31	0.74
**Composite Splint**	100N at 0°	0.3	0.33	0.18	0.44
	100N at 45°	0.19	0.24	0.45	0.62
**FRC Splint**	100N at 0°	0.21	0.25	0.17	0.36
	100N at 45°	0.13	0.19	0.38	0.41
**Metal Wire Splint**	100N at 0°	0.19	0.21	0.25	0.34
	100N at 45°	0.26	0.25	0.36	0.51
**Peek Splint**	100N at 0°	0.08	0.16	0.37	0.25
	100N at 45°	0.17	0.29	0.59	0.54

The composite splint showed stress values at 0° of 0.3, 0.33, 0.18, and 0.44 MPa for the PDL of central incisors, lateral incisors, canine teeth, and cortical bone, respectively. Under 45° loading, the PDL of canine teeth exhibited a significant increase in stress to 0.45 MPa, indicating potential stress concentration in this area. The fiber-reinforced composite (FRC) splint exhibited reduced stress values across the board, with the PDL of central incisors, lateral incisors, canine teeth, and cortical bone registering 0.21, 0.25, 0.17, and 0.36 MPa, respectively, under a 0° load. At 45°, the stress values decreased, highlighting the effective stress distribution capability of the FRC splint.

The metal wire splint showed stress values under a 0° load of 0.19, 0.21, 0.25, and 0.34 MPa for the PDL of central incisors, lateral incisors, canine teeth, and cortical bone, respectively. This splint demonstrated moderate stress reduction capabilities, with increased stress at 45°, particularly for canine teeth and cortical bone. The PEEK splint exhibited the lowest stress values under a 0° load, with values of 0.08, 0.16, 0.37, and 0.25 MPa for the PDL of the central incisors, lateral incisors, canine teeth, and cortical bone, respectively. At 45°, stress values increased notably in the PDL of canine teeth (0.59 MPa), suggesting that PEEK may be most effective at distributing stress under a 0° load. Overall, the PEEK and FRC splints demonstrated superior performance in minimizing stress across dental structures compared to other splint materials, especially under straight load conditions.

**Fig. 2:**
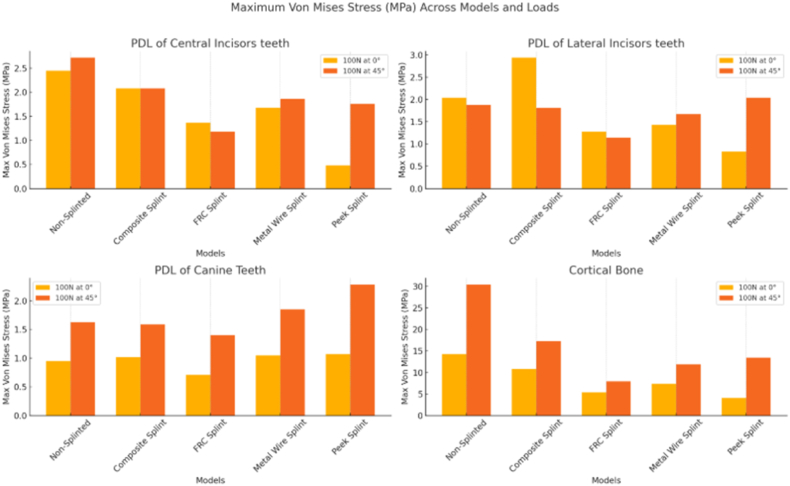
Bar charts illustrating the maximum Von Mises stress (MPa) for each dental part across different splint models and load conditions.

Table 2 presents the maximum Von Mises stress, in MPa, observed in different models and loading conditions, highlighting the peak stress levels in the periodontal ligament and cortical bone. In the non-splinted model, the PDL of central incisors, lateral incisors, canine teeth, and cortical bone experienced maximum stress of 2.45, 2.04, 0.95, and 14.27 MPa, respectively, at a 0° load. At 45°, the stress in the cortical bone to 30.38 MPa, indicating a potential risk for structural damage (Table 2, Fig. 2).

**Table 2:** Maximum von mises stress (MPa).

Model	LOAD (N)	PDL of Central Incisors teeth	PDL of Lateral Incisors teeth	PDL of Canine Teeth	Cortical Bone
**Non-Splinted**	100N at 0°	2.45	2.04	0.95	14.27
	100N at 45°	2.72	1.88	1.63	30.38
**Composite Splint**	100N at 0°	2.08	2.94	1.02	10.81
	100N at 45°	2.08	1.81	1.59	17.31
**FRC Splint**	100N at 0°	1.37	1.28	0.71	5.42
	100N at 45°	1.18	1.14	1.4	7.96
**Metal Wire Splint**	100N at 0°	1.68	1.43	1.05	7.39
	100N at 45°	1.86	1.67	1.85	11.9
**Peek Splint**	100N at 0°	0.48	0.83	1.07	4.06
	100N at 45°	1.76	2.04	2.28	13.45

**Fig. 3:**
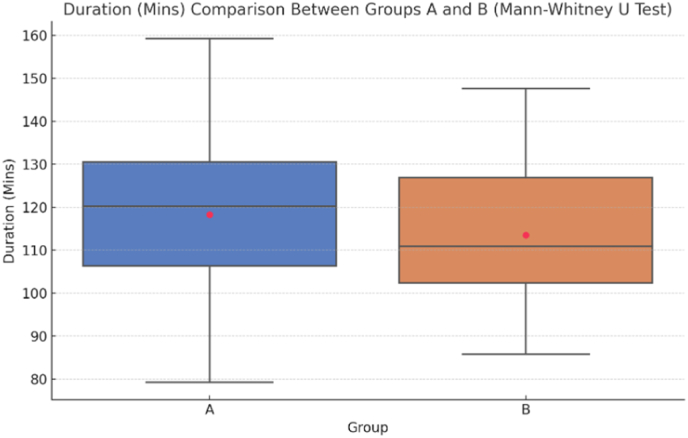
Mann Whitney *U* Test comparing groups A &B.

Table 3 presents the results of the Mann-Whitney *U* test comparing the duration of the two groups, A and B, each consisting of 22 samples. Group A had a mean duration of 114.14 min with a standard deviation of 15.462, while Group B had a mean duration of 113.23 min and a standard deviation of 15.316. The mean ranks for Groups A and B were 22.84 and 22.16, respectively, with a sum ranks of 502.50 and 487.50. The median for Group A 113, whereas that for Group B was 116.5. The Mann-Whitney U value was calculated to be 234.500, with a Z value of −0.177. The p-value obtained was 0.860, indicating no statistically significant difference in the duration between the two groups. The high p-value, which is greater than 0.05, suggests that the null hypothesis, which posits no difference between the groups, cannot be rejected (Table 3, Fig. 3).

**Table 3:** Mann-whitney *U* test for duration (mins).

Group	N	Mean	Std.Deviation	Mean Rank	Sum of ranks	Median	Mann-Whitney U Value	Z value	P-Value the Mann-Whitney U Value
Duration (Mins) A	22	114.14	15.462	22.84	502.50	113	234.500	−0.177	0.860#
Duration (Mins) B	22	113.23	15.316	22.16	487.50	116.5

**Fig. 4:**
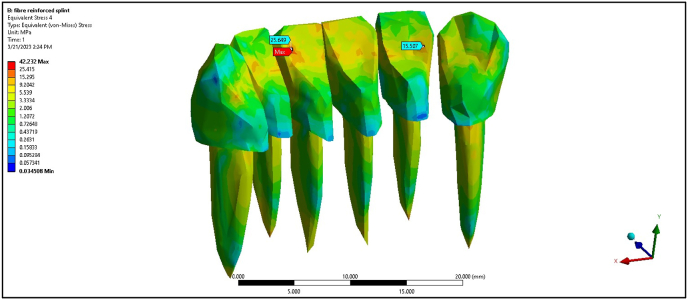
Maximum von Mises stress on fibre reinforced composite splint at 0°.

**Fig. 5:**
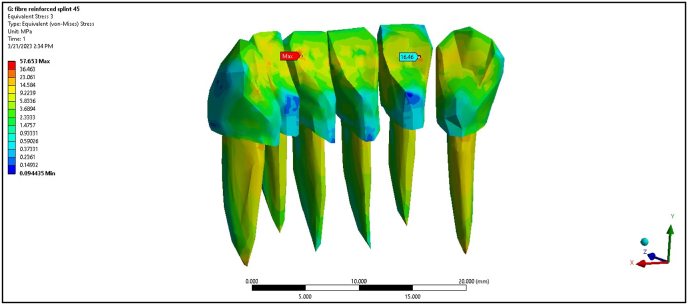
Maximum von Mises stress on Fibre reinforced composite splint at 45°.

The composite splint registered maximum stress values at 0° of 2.08, 2.94, 1.02, and 10.81 MPa for the PDL of central incisors, lateral incisors, canine teeth, and cortical bone, respectively. The composite splint showed a substantial decrease in cortical bone stress compared with the non-splinted model, although the lateral incisors experienced higher stress. At 45°, the cortical bone stress was 17.31 MPa. The FRC splint significantly reduced maximum stress values, with readings of 1.37, 1.28, 0.71, and 5.42 MPa at 0° load (Fig. 4). At 45°, the maximum stress values slightly increased but remained lower than those in the non-splinted and composite models, demonstrating the capability of the FRC splint to distribute stress effectively (Fig. 5).

**Fig. 6:**
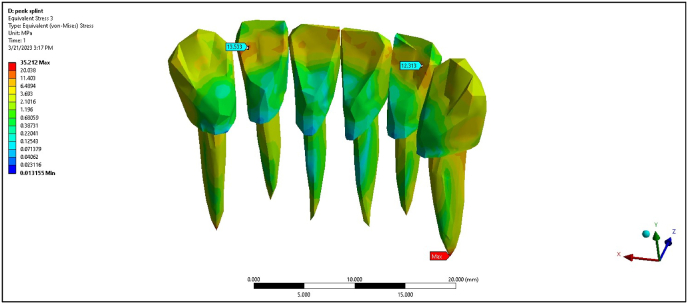
Maximum von Mises stress on PEEK splint at 0°.

**Fig. 7:**
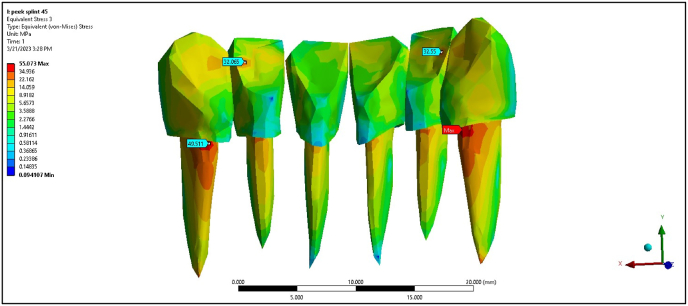
Maximum von Mises stress on PEEK splint at 45°.

The metal wire splint under a 0° load showed maximum stress values of 1.68, 1.43, 1.05, and 7.39 MPa for the PDL of central incisors, lateral incisors, canine teeth, and cortical bone, respectively (Table 2). At 45°, the metal wire splint exhibited increased stress in all areas, with the cortical bone experiencing 11.9 MPa, which is lower than the non-splinted scenario but higher than the FRC splint. The PEEK splint provided the lowest maximum stress values at 0° load, with the PDL of the central incisors, lateral incisors, canine teeth, and cortical bone at 0.48, 0.83, 1.07, and 4.06 MPa, respectively (Fig. 6). At 45°, the maximum stress for the PDL of canine teeth increased to 2.28 MPa, but the PEEK splint remained effective in distributing stress more evenly than the other models (Fig. 7).

## 4 Discussion

This study assessed the stress distribution in periodontally compromised teeth splinted with various materials including composite, fiber-reinforced composite (FRC), polyetheretherketone (PEEK), and metal. The findings revealed significant differences in how these materials manage stress, which is critical for understanding their effectiveness in clinical applications. Specifically, this study employed finite element analysis (FEA) to simulate the biomechanical environment of the oral cavity, focusing on how each material affects stress distribution under conditions mimicking those found in patients with severe periodontal disease. Periodontal compromise, characterized by substantial bone loss, presents a challenge for maintaining tooth stability and function. The choice of splint material plays a pivotal role in addressing these challenges by influencing the mechanical forces acting on teeth and surrounding structures.

Finite element analysis revealed distinct variations in the stress distribution across different splint materials. PEEK and FRC splints showed superior performance in minimizing stress concentrations across dental structures, particularly under straight load conditions. This finding aligns with those of previous studies that have highlighted the mechanical advantages of FRC and PEEK in dental applications owing to their high strength and favorable biomechanical properties.^11^^,^^12^

Composite materials, although popular for their ease of use, demonstrated higher stress values than FRC and PEEK. This outcome may be attributed to the lower stiffness and strength of the composite materials, which can lead to increased stress concentrations, particularly under oblique loading conditions. This observation is consistent with other studies that reported similar stress patterns in composite-based splints.^13^^,^^14^

Metal splints, traditionally considered the gold standard owing to their superior mechanical properties, exhibit moderate stress-reduction capabilities. However, their lack of flexibility and potential for corrosion are well-documented limitations that can affect the long-term outcomes of periodontal splinting.^15^^,^^16^ The results suggest that while metal splints provide adequate stress distribution, alternative materials, such as PEEK and FRC, offer better adaptability and reduced stress concentrations.^17^

Several studies have evaluated the effectiveness of various splint materials in managing the stress distribution in periodontally compromised teeth. Karbhari et al. demonstrated that FRC materials significantly reduced stress concentrations in splinted teeth compared with conventional composites.^18^ Our findings corroborate this evidence, highlighting the enhanced stress distribution capabilities of the FRC materials.

In contrast, studies of PEEK splints have shown mixed results. While some research indicates that PEEK offers excellent biocompatibility and mechanical properties, other studies suggest that its clinical application in splinting is limited owing to its higher cost and processing challenges.^17^ Nonetheless, our study supports the potential of PEEK as a viable alternative to traditional splint materials, particularly given its superior performance under straight loading conditions.

The observed differences in the stress distribution between the materials can also be attributed to their inherent mechanical properties, such as Young's modulus and density. For example, the higher Young's modulus of PEEK and FRC allows for better stress distribution, reducing the risk of stress concentration and potential failure.^19^ This mechanical advantage is crucial in periodontally compromised teeth, where even minor stress concentrations can exacerbate bone loss and tooth mobility.

The choice of splint material in periodontal therapy should consider not only the mechanical properties, but also the clinical scenario and patient-specific factors. The findings of this study suggest that PEEK and FRC may offer superior stress distribution, making them suitable choices for patients with severe periodontal compromise. These materials can potentially improve the longevity of splinted teeth by minimizing stress concentration and reducing the risk of further periodontal damage.

Furthermore, the use of FRC and PEEK splints could facilitate better patient outcomes by enhancing patient comfort and esthetics. Their adaptability and ability to mimic the natural tooth structure make them appealing options for patients concerned with the visual appearance of their splints. The biocompatibility of PEEK in particular adds another advantage: it reduces the risk of adverse tissue reactions and improves patient compliance.^20^

The strengths of this study lie in its robust methodological approach and comprehensive analysis of splint materials for periodontally compromised teeth. Finite element analysis (FEA) allows for detailed simulations of stress distribution under various loading conditions, providing insights that are challenging to capture experimentally. By evaluating a range of materials, including composites, fiber-reinforced composites (FRC), polyetherether ketone (PEEK), and metal, the study offered a thorough comparison of their mechanical properties and effectiveness. The inclusion of both vertical and oblique loading scenarios further enhances the clinical relevance of the findings. Additionally, the focus on periodontally compromised teeth with significant bone loss addresses a critical area in periodontal therapy, potentially guiding clinicians in choosing the most suitable splint material to improve patient outcomes.

Although this study provides valuable insights into the stress distribution of different splint materials, it is important to acknowledge certain limitations. The finite element models used in this study were based on idealized conditions that may not fully replicate the complex biomechanics of the oral environment. Future studies should incorporate more comprehensive models that account for variations in bone density, periodontal ligament properties, and patient-specific anatomical differences.

Additionally, clinical studies are needed to validate the findings of finite element analysis. Long-term clinical trials comparing the performance of different splint materials in real-world scenarios would provide more definitive evidence for their effectiveness and durability. Such research would be instrumental in establishing standardized guidelines for selecting appropriate splint materials for periodontal therapy.

## 5 Conclusion

Selecting the appropriate splint material plays a critical role in managing periodontally compromised teeth. Among the materials analyzed, PEEK and fiber-reinforced composite (FRC) demonstrated superior stress distribution properties compared to traditional composites and metals. These advanced materials effectively minimize stress concentrations while enhancing biomechanical stability, which can significantly contribute to better clinical outcomes. By reducing the mechanical strain on the periodontal ligament and cortical bone, PEEK and FRC splints have the potential to prolong the survival of splinted teeth, making them valuable alternatives in periodontal therapy.

## Patient consent

Patient consent was obtained.

## Ethical clearance

Ethical clearance was obtained from the Institutional Review Board.

## Funding

SELF FUNDED PROJECT.

## Declaration of competing interest

The authors declare that there is no conflict of interest.
